# Pharmacological treatment of chronic tic disorders

**DOI:** 10.1192/j.eurpsy.2026.11779

**Published:** 2026-07-31

**Authors:** T. Gutierrez Higueras

**Affiliations:** Child and Adolescent Psychiatry, Servicio Andaluz de Salud, Jaén, Spain

## Abstract

**Introduction:**

Chronic tic disorders, and persistent motor or vocal disorders, are neuropsychiatric conditions characterized by repetitive, sudden, and arrhythmic movements or vocalizations that persist for more than a year. Pharmacological treatment has long been primarily with antipsychotics, but the adverse effects induced by these drugs have prompted the search for safer and more effective alternatives.

**Objectives:**

To evaluate the efficacy of pharmacological treatments for reducing the severity of tics in individuals with chronic tic disorders, as well as to describe the risks associated with such treatments.

**Methods:**

A literature review was conducted in Pubmed using keywords such as “chronic tic disorders” and “treatments”, published between 2015 and 2025.

**Results:**

Significant evidence was found for reducing tic intensity with haloperidol, risperidone, aripiprazole, tiapride, and clonidine. Drugs with less evidence for reducing tic intensity include pimozide, ziprasidone, metoclopramide, guanfacine, and topiramate. Significant risks were identified, including weight gain and hyperprolactinemia (primarily risperidone), sedation (clonidine and guanfacine), and extrapyramidal symptoms (pimozide and haloperidol).

**Conclusions:**

Various pharmacological treatments can help reduce the severity of chronic tics. Haloperidol, risperidone, aripiprazole, tiapride, and clonidine show the most evidence of efficacy, while other drugs such as pimozide, ziprasidone, and guanfacine have limited support. Treatment selection should balance efficacy and risks, including weight gain, hyperprolactinemia, sedation, and extrapyramidal symptoms, within a multidisciplinary and shared decision-making approach.

**Disclosure of Interest:**

None Declared

